# Intensity-Modulated Radiation Therapy with Stereotactic Body Radiation Therapy Boost for Unfavorable Prostate Cancer: A Report on 3-Year Toxicity

**DOI:** 10.3389/fonc.2017.00005

**Published:** 2017-02-07

**Authors:** Ima Paydar, Abigail Pepin, Robyn A. Cyr, Joseph King, Thomas M. Yung, Elizabeth G. Bullock, Siyuan Lei, Andrew Satinsky, K. William Harter, Simeng Suy, Anatoly Dritschilo, John H. Lynch, Thomas P. Kole, Sean P. Collins

**Affiliations:** ^1^Department of Radiation Medicine, Georgetown University Hospital, Washington, DC, USA; ^2^George Washington University, Washington, DC, USA; ^3^University of South Carolina School of Medicine, Columbia, SC, USA; ^4^Department of Urology, Georgetown University Hospital, Washington, DC, USA; ^5^Department of Radiation Oncology, The Valley Hospital, Ridgewood, NJ, USA

**Keywords:** prostate cancer, SBRT, IMRT, CyberKnife, common terminology criteria

## Abstract

**Background:**

Recent data suggest that intensity-modulated radiation therapy (IMRT) plus brachytherapy boost for unfavorable prostate cancer provides improved biochemical relapse-free survival over IMRT alone. Stereotactic body radiation therapy (SBRT) may be a less invasive alternative to brachytherapy boost. Here, we report the 3-year gastrointestinal (GI) and genitourinary (GU) toxicities of IMRT plus SBRT boost.

**Materials and methods:**

Between March 2008 and September 2012, patients with prostate cancer were treated with robotic SBRT (19.5 Gy in three fractions) followed by fiducial-guided IMRT (45–50.4 Gy) on an institutional protocol. Toxicity was prospectively graded using the common terminology criteria for adverse events version 4.0 (CTCAEv.4) at the start of and at 1- to 6-month intervals after therapy. Rectal telangiectasias were graded using the Vienna Rectoscopy Score (VRS).

**Results:**

At a median follow-up of 4.2 years (2.4–7.5), 108 patients (4 low-, 45 intermediate-, and 59 high-risk) with a median age of 74 years (55–92) were treated with SBRT plus IMRT, with 8% on anticoagulation and an additional 48% on antiplatelet therapy at the start of therapy. The cumulative incidence of late ≥grade 2 GI toxicity was 12%. Of these, 7% were due to late rectal bleeding, with six patients requiring up to two coagulation procedures. One patient with rectal telangiectasias was treated with hyperbaric oxygen (grade 3 toxicity). No rectal fistulas or stenoses were observed. Ten patients had multiple non-confluent telangiectasias (VRS grade 2), and three patients had multiple confluent telangiectasias (VRS grade 3). The cumulative incidence of late grade 3 GU toxicity was 6%. Most late toxicities were due to hematuria requiring bladder fulguration. There were no late ≥grade 4 GU toxicities.

**Conclusion:**

Rates of clinically significant GI and GU toxicities are modest following IMRT plus SBRT boost. Future studies should compare cancer control, quality of life, and toxicity with other treatment modalities for patients with high-risk prostate cancer.

## Introduction

Prostate cancer is the most common malignancy in men in the United States, with an estimated 220,800 men diagnosed in 2015 (1). Of these patients, approximately 15% present with high-risk disease (2). Radiotherapy is the mainstay for treatment of such patients, and several randomized prospective trials have demonstrated that dose-escalated radiotherapy results in improved biochemical-free survival (3–5). Further improvements have also been achieved with the advent of image-guided radiation therapy (IGRT) (6) and low dose rate (LDR) brachytherapy boost (7, 8).

Recent clinical data have demonstrated that large radiation fraction sizes likely confer a radiobiologic advantage in the setting of prostate adenocarcinoma (9), thus providing the rationale for high dose rate (HDR) brachytherapy as a boost to external beam radiation therapy (EBRT) for intermediate- and high-risk patients. Several institutional series have reported favorable outcomes, with biochemical control rates of 87–88 and 69% at 5–7 years for intermediate- and high-risk disease, respectively (10–13). These results have subsequently been confirmed in randomized trials (14, 15). Not surprisingly, such cancer control outcomes present with an increased risk of clinically significant long-term genitourinary (GU) toxicities such as urethral stricture (16–18).

In an effort to maximize the benefit of administering high doses per fraction and patient acceptance, we have examined the use of stereotactic body radiation therapy (SBRT) as a prostatic boost to image-guided intensity-modulated radiation therapy (IMRT) for the treatment of patients with unfavorable clinically localized prostate cancer. Previously, we reported early outcomes of this treatment modality, with a 3-year biochemical-free survival rate of 100% for intermediate-risk and 89.8% for high-risk disease (19). Similarly, we reported that such a therapy conferred minimal impact on long-term quality of life (QOL) (19). Several other studies have supported our early results (20–23). Here, we report the 3-year gastrointestinal (GI) and GU toxicity from this therapy.

## Materials and Methods

### Patient Selection

Patients with histologically confirmed adenocarcinoma of the prostate were included in the study. Exclusion criteria included clinically involved lymph nodes, bone metastases, or prior pelvic radiotherapy. Androgen deprivation therapy (ADT) was considered for all intermediate- and high-risk patients and ultimately was administered at the discretion of the treating physicians. The MedStar Health Research Institute-Georgetown University Oncology Institutional Review Board approved this study. This research study was carried out under a continuing review approved by this Institutional Review Board (IRB#2009-510). Continuing review is in accordance with institutional guidelines and was approved though expedited review by the IRB Chair or designee on 1/8/2016. The informed consent requirement was waived by the Committee that approved the study, and all data used in this study were anonymized.

### SBRT Treatment Planning and Delivery

All patients had four or more gold fiducials placed in the prostate prior to treatment planning. To allow for fiducial stabilization, planning images were obtained a minimum of 7 days after fiducial placement. Patients underwent magnetic resonance imaging (MRI) followed shortly thereafter by a thin cut (1.25 mm) CT scan. For the few patients with contraindications to MRI, CT-urethrogram was employed as an alternative imaging approach to identify the location of the prostatic apex (24). Both scans were performed with an empty bladder. Patients were advised to adhere to a low-gas, low-motility diet starting at least 5 days prior to all treatment planning imaging and treatment delivery. They were also instructed to remain nothing by mouth (Non-Per *Os*) for at least 4 hours prior to imaging as well as SBRT treatment. An enema was administered 1–2 hours prior to imaging and SBRT treatment.

CT and MR images were fused for treatment planning. The clinical target volume (CTV) included the prostate, areas of radiographic extracapsular extension (ECE), and the proximal seminal vesicles to the point of separation. Pelvic lymph nodes were not included in the CTV. The SBRT planning target volume 1 (PTV1) equaled the CTV expanded 3 mm posteriorly and 5 mm in all other dimensions. The prescription dose was 19.5 Gy to the PTV1 delivered in three fractions of 6.5 Gy over 3–5 days. The prescription isodose line was limited to ≥75%, which limited the maximum prostatic urethra dose to 133% of the prescription dose. The rectum, bladder, penile bulb, and membranous urethra were contoured and evaluated with dose–volume histogram (DVH) analysis during treatment planning using Multiplan (Accuray Inc., Sunnyvale, CA, USA) inverse treatment planning. Less than 1 cc of the rectum and less than 10 cc of the bladder were to receive 19.5 Gy. Less than 50% of the membranous urethra was to receive 18 Gy. Further details on dose and volume constraints to the critical structures have been previously described (19, 25).

### Intensity-Modulated Radiation Therapy

Patients initiated IMRT treatment the week following SBRT. The more generous planning target volume 2 (PTV2) included a margin of 1.0 cm around the CTV except at the rectal interface where a margin of 0.5 cm was added. Daily doses of 1.8 Gy were delivered to the PTV2 5 days a week to a total dose of 45–50.4 Gy in 25–28 fractions. One-hundred percent of the PTV2 was to receive at least 95% of the prescription dose, and 5% of the volume was to receive no more than 105% of the prescription dose. For the bladder and rectum, the maximum dose constraint limit was 50 Gy, the full-volume dose constraint limit was 30 Gy, and no part of either volume received more than 55.5 Gy. Dose to the femoral heads was limited to 45 Gy.

### Linear-Quadratic Transformation of a Sample Combined Physical IMRT Plus SBRT Boost DVH to a Radiobiologically Equivalent DVH

**Figure 1:**
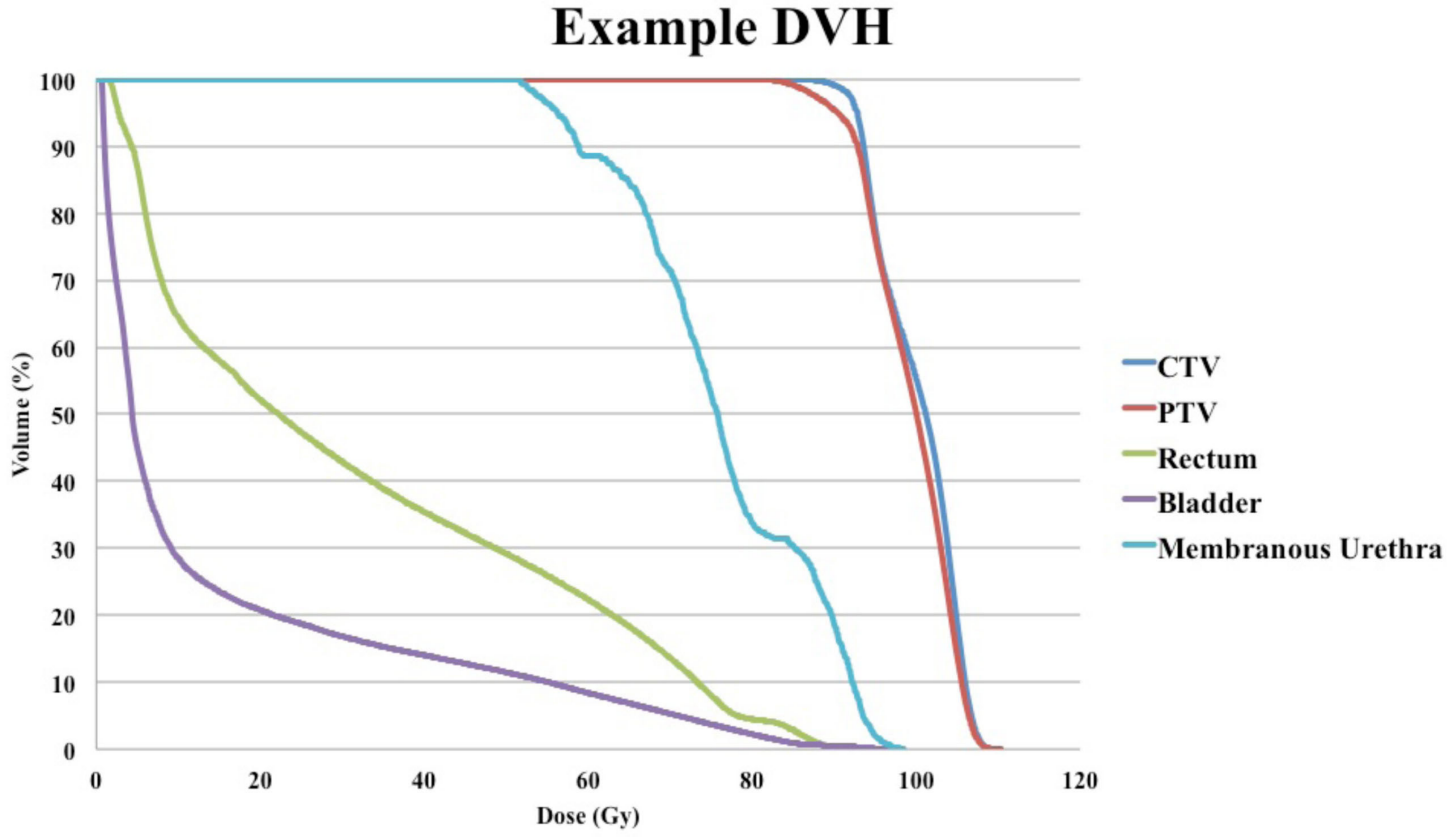
**Example of radiobiologically equivalent dose–volume histogram (DVH) of a patient with late grade 2 rectal bleeding treated with two argon plasma coagulations and no genitourinary toxicity**.

A radiobiologically equivalent dose DVH was generated by adding doses in 2 Gy equivalents for IMRT and SBRT plans from a sample patient (26). Cumulative DVHs were extracted from the treatment planning software and converted to radiobiologically equivalent DVHs using MIM software (MIMvista Corporation, Cleveland, OH, USA). An α/β ratio of 1.5 was utilized to transform target volume doses (CTV and PTV), and an α/β ratio of 3 was used to transform doses for all other organs at risk. Combination of radiobiologically equivalent DVH for an example patient is shown in Figure 1.

### Follow-up and Toxicity Assessment

Patients were assessed at the start of and at 1 month after therapy, every 3 months for the first year, and every 6 months thereafter. The utilization of alpha-antagonists, oral corticosteroids, anticoagulation or antiplatelet therapy, and anti-diarrheal therapy was documented at each visit. GI and GU toxicities were prospectively documented at follow-up visits using the National Cancer Institute Common Terminology Criteria (CTC) version 4.0 as previously described (27). Specifically, acute toxicity was defined as occurring up to the first 6 months after therapy, and late toxicity was defined as occurring at 6 months or thereafter.

Grade 1 rectal bleeding was defined as transient and not requiring medications for symptomatic management. Grade 2 rectal bleeding represented bleeding which required a new medication (i.e., steroid suppository) or up to two argon plasma coagulations (APCs). More than two APC procedures, a blood transfusion, or use of hyperbaric oxygen (HBO) was defined as grade 3 rectal bleeding. Grade 1 diarrhea was defined as transient diarrhea not requiring medical management. Grade 2 diarrhea was defined as increased stool frequency requiring management with anti-diarrheal medication. The development of rectal strictures or fistulas was defined as grade 4 GI toxicity. Furthermore, because all patients were treated on an institutional protocol, all rectal bleeding events were assessed by endoscopy. Radiation-induced rectal telangiectasias were graded using the Vienna Rectoscopy Score (VRS), with grade 1 defined as a single telangiectasia, grade 2 as multiple non-confluent telangiectasias, and grade 3 as multiple confluent telangiectasias (28).

For GU toxicities, a transient toxicity requiring no new medications for symptomatic management was considered grade 1. The use of a new medication or an increase in the dosage of an already-used medication for symptomatic management was considered grade 2. Grade 3 hematuria, urethral stricture, and urinary retention were defined as requiring an outpatient procedure such as fulguration, urethral dilation, or transurethral resection of the prostate (TURP), respectively. Any toxicity requiring initiation of more invasive therapy was defined as grade 4.

### Statistical Analysis

Logistic regression analysis was performed to identify patient characteristics associated with an increased risk of late ≥grade 2 GI toxicity, late ≥grade 2 rectal bleeding, and late grade 3 GU toxicity. Statistical analysis was performed using MedCalc software (Ostend, Belgium).

## Results

From March 2008 to September 2012, 108 prostate cancer patients were treated on an institutional IMRT plus SBRT boost protocol. The median follow-up was 4.2 years (range 2.4–7.5). Patient characteristics are shown in Table 1. The median age was 74 years (range 55–91). Similar numbers of Caucasians (47%) and African-Americans (42%) were treated. Patients were generally healthy, with a Charlson comorbidity index of 0–1 in 75%. Eight percent were on anticoagulation therapy, and an additional 48% were on antiplatelet therapy at the start of radiation therapy. The median pre-treatment prostate-specific antigen was 9.1 ng/ml (range 0.86–39.8 ng/ml). By D’Amico classification, 4% were diagnosed with low-, 42% with intermediate-, and 55% with high-risk disease. Seventy-eight percent of patients were treated with an IMRT dose of 45 Gy in 25 fractions. Sixty-three percent received ADT for a median of 6 months (range 3–36 months).

**Table 1:** **Patient characteristics and treatment specifics**.

	Percent patients (*n* = 108)
**Age (years): median 74 (55–91)**	
<60	6
60–69	24
70–79	52
≥80	19

**Race**	
White	47
Black	42
Hispanic	3
Asian	2
Other	6

**Pre-Tx prostate-specific antigen (ng/ml): median 9.1 (0.86–39.8)**	
≤10	51
>10 and ≤20	34
>20	15

**T stage**	
T1c	46
T2a	12
T2b	28
T2c	13
T3	1

**Gleason score**	
6	9
7	51
8	25
9	15

**Charlson comorbidity index**	
0–1	75
2–3	23
4	3

**Risk group (D’Amico)**	
Low	4
Intermediate	42
High	55

**Hormone therapy**	
Yes	63
No	37

**Anti-coagulation/-platelet therapy**	
Anticoagulation	8
Antiplatelet	48

**Intensity-modulated radiation therapy dose**	
45 Gy	78
50.4 Gy	19
Other	3

**Figure 2:**
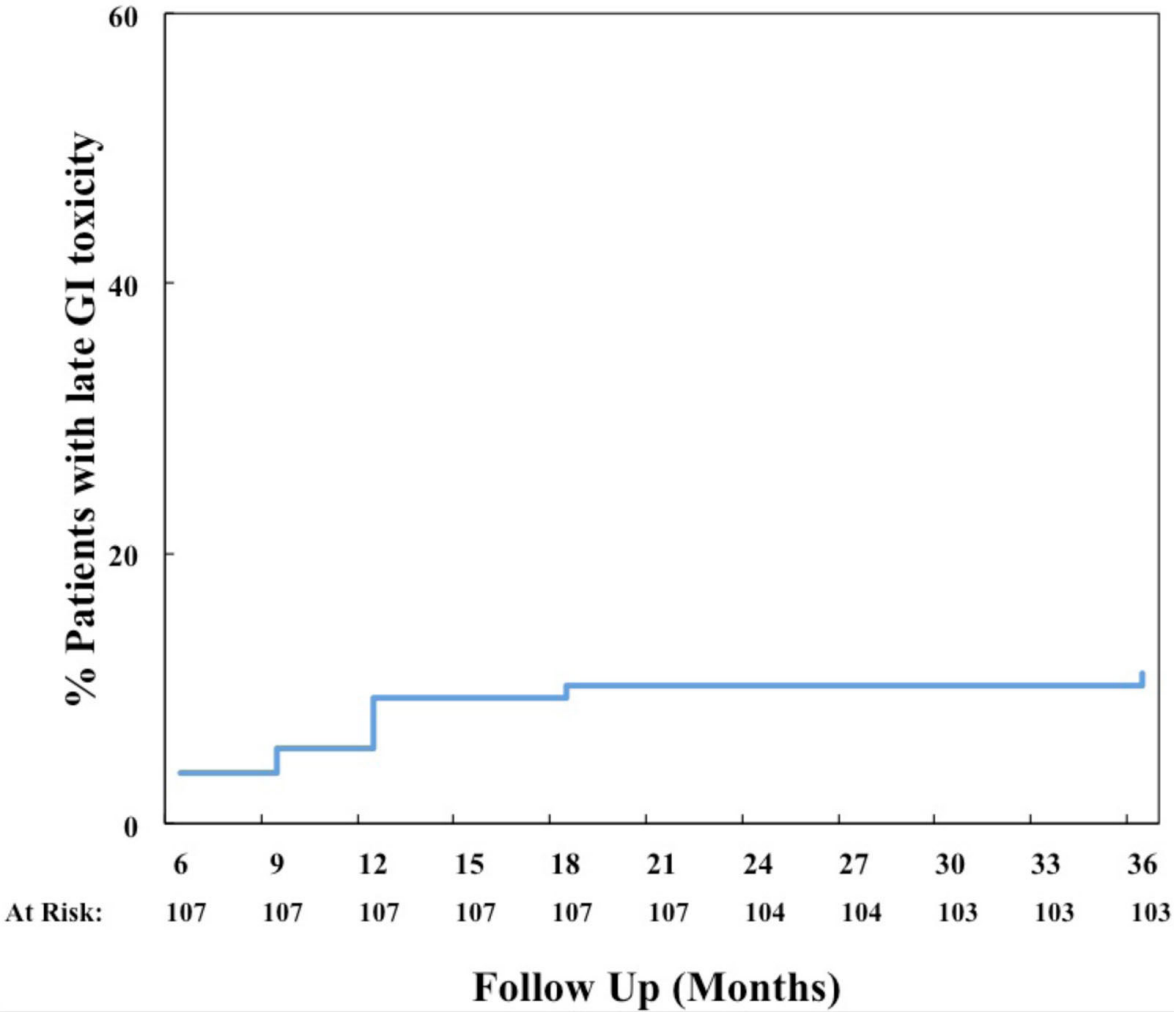
**Cumulative late ≥grade 2 gastrointestinal (GI) toxicity**.

The prevalence of GI and GU toxicities following treatment are shown in Tables 2 and 3. The majority of toxicities were observed at one specific follow-up appointment and resolved on subsequent follow-ups. The most common acute grade 2 GI toxicity was diarrhea, with a peak in 7% of patients at 1 month (Table 2). There were no acute ≥grade 2 rectal bleeding events. The cumulative rate of late ≥grade 2 GI toxicity was 12% (Figure 2), 7% of which was due to rectal bleeding and 5% due to diarrhea. Rectal bleeding occurred most commonly at 12 months following radiation therapy. Of the seven patients who developed ≥grade 2 bleeding, one was on both anticoagulation and antiplatelet therapy, three on antiplatelet therapy only, and three on neither therapy. Six of these patients were assigned as grade 2 bleeding for requiring one or two coagulation procedures, and one was assigned as grade 3 for undergoing HBO. Further details for patients who developed rectal bleeding are shown in Table 4. Of note, logistic regression analysis identified no patient characteristics associated with an increased risk of late ≥grade 2 GI toxicity or late ≥grade 2 rectal bleeding, including use of anticoagulation or antiplatelet therapy (data not shown).

**Table 2:** **Prevalence of CTC graded gastrointestinal (GI) toxicities at each follow-up to 36 months**.

	Month	1	3	6	9	12	18	24	30	36

Toxicity	Grade	%	%	%	%	%	%	%	%	%
Diarrhea	0	75	82	83	74	79	95	78	77	79
	1	19	14	13	23	19	5	20	22	21
	2	7	3	4	3	2	0	2	1	0

Proctitis	0	90	96	95	95	93	96	95	96	93
	1	10	4	5	5	7	4	5	4	7
	2	0	0	0	0	0	0	0	0	0

Rectal bleeding	0	87	96	87	85	83	84	81	87	87
	1	13	4	13	15	12	14	18	13	12
	2	0	0	0	0	4	2	1	0	1
	3	0	0	0	0	1^a^	0	0	0	0

Highest GI	0	62	76	72	66	64	68	65	68	68
	1	31	21	24	31	31	29	32	31	31
	2	7	3	4	3	4	3	3	1	1
	3	0	0	0	0	1	0	0	0	0

*^a^Patient with non-healing ulcer required hyperbaric oxygen and was assigned as grade 3 toxicity*.

**Table 3:** **Prevalence of CTC graded genitourinary (GU) toxicities at each follow-up to 36 months**.

	Month	1	3	6	9	12	18	24	30	36

Toxicity	Grade	%	%	%	%	%	%	%	%	%
Hematuria	0	97	98	96	97	95	92	94	95	93
	1	3	2	4	3	5	6	5	3	1
	2	0	0	0	0	0	0	0	0	4
	3	0	0	0	0	0	2	1	1	1

Dysuria	0	88	91	91	91	86	95	93	92	93
	1	12	9	9	9	12	5	6	7	7
	2	0	0	0	0	2	0	1	1	0

Incontinence	0	77	85	86	80	82	83	79	68	77
	1	21	13	14	18	16	16	21	29	19
	2	2	2	0	2	2	1	0	3	4

Urinary frequency/urgency	0	53	58	73	71	69	69	70	51	51
	1	45	41	27	28	28	30	29	50	49
	2	2	1	0	1	3	1	1	0	0

Retention	0	62	69	71	66	71	67	62	56	59
	1	23	23	18	23	16	20	23	34	32
	2	15	8	10	11	12	12	14	10	9
	3	1	0	1	0	0	1	0	0	0

Highest GU	0	26	37	48	42	46	48	40	34	39
	1	55	53	40	44	38	36	45	52	44
	2	18	10	10	15	16	14	14	12	16
	3	1	0	1	0	0	3	1	1	1

**Table 4:** **Patients with late ≥grade 2 rectal bleeding**.

Patient	Age	Antiplatelets	Anticoagulants	Time to bleed (months)	Vienna Rectoscopy Score grade	Argon plasma coagulation
1	72	N/A	N/A	12, 18, and 24	2	Yes
2	79	Aspirin (unknown dose)	N/A	12	2	Yes
3	82	Aspirin (81 mg)	N/A	12	3	Yes
4^a^	69	Aspirin (81 mg)	N/A	12	2	No
5	75	Aspirin (325 mg)	Apixaban	12	2	Yes
6	66	N/A	N/A	18	3	Yes
7	65	N/A	N/A	36	2	Yes

*^a^One patient with a non-healing rectal ulcer elected to have treatment with hyperbaric oxygen and was assigned as grade 3*.

A total of 42 patients underwent a colonoscopy during the follow-up period either to assess the etiology of rectal bleeding or for routine cancer screening. Radiation-induced telangiectasias were noted in 16 cases and were graded as VRS grade 1 in 3 patients, grade 2 in 10 patients, and grade 3 in 3 patients. One patient was noted to have an incidental grade 1 ulcer, which spontaneously resolved on subsequent colonoscopy. Importantly, no rectal strictures or fistulas (grade 4 toxicity) were noted.

**Figure 3:**
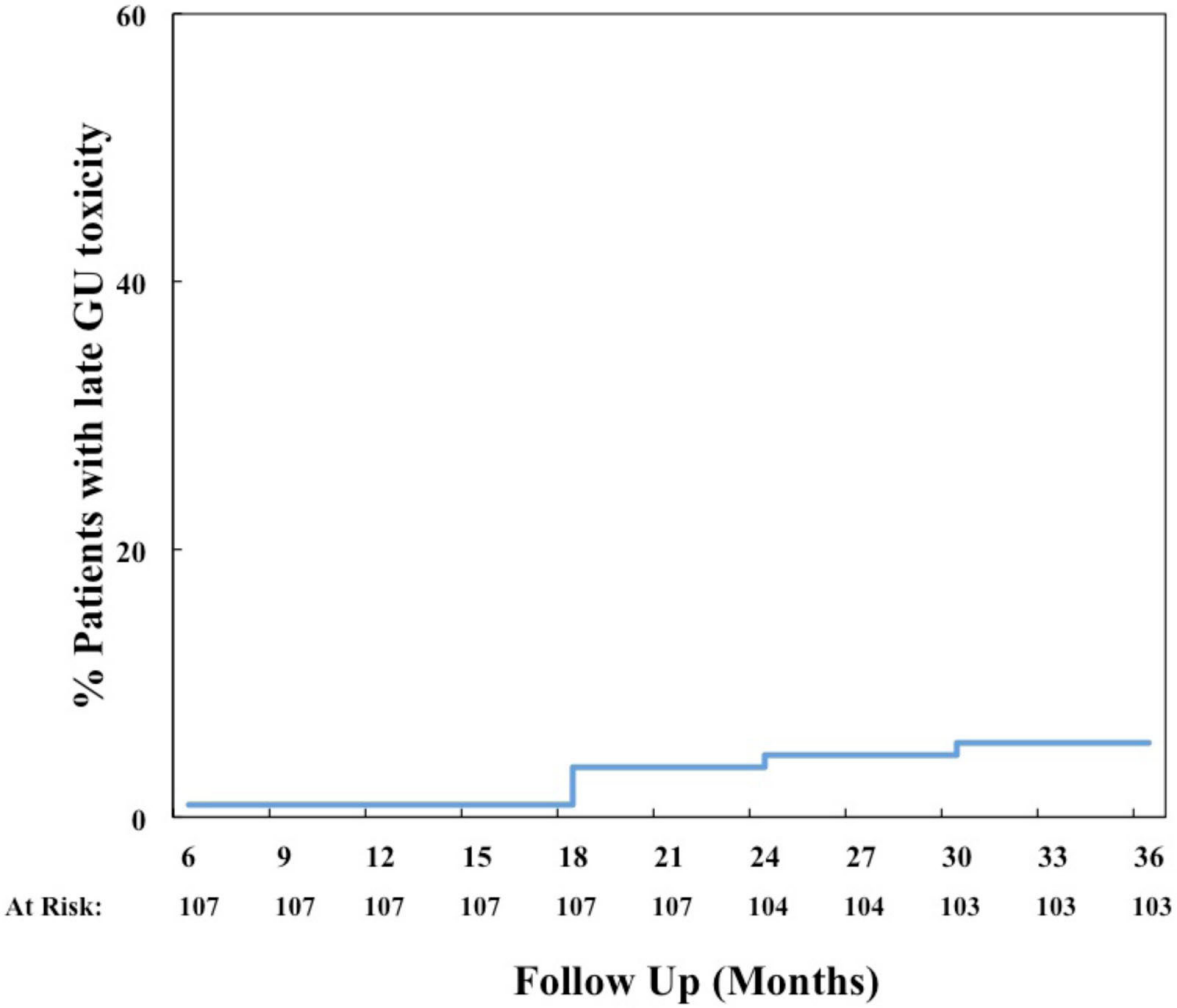
**Cumulative late ≥grade 3 genitourinary (GU) toxicity**.

The most common acute grade 2 GU toxicity was urinary retention relieved by medical management, peaking at 1 month (Table 3). One patient with acute urinary retention underwent a TURP and was classified as grade 3. There were no ≥grade 2 hematuria events acutely. The cumulative rate of late ≥grade 2 GU toxicity was 40%. The majority of these toxicities were due to obstructive or irritative symptoms requiring medical management with alpha-antagonists and/or anti-muscarinics. In fact, the cumulative rate of late ≥grade 3 GU toxicity was much lower at 6% (Figure 3), 4% due to hematuria and 2% due to retention. The most common areas of radiation cystitis noted on cystoscopy were the bladder neck, trigone, or lateral walls. Of the four patients who experienced bleeding, two were on antiplatelet therapy and none were on anticoagulation therapy. One of these patients with recurrent bleeding secondary to vigorous physical activity also elected to proceed with HBO. Details for patients with hematuria are provided in Table 5. Of the two patients who underwent a TURP for late urinary retention, one had a long history of benign prostatic hypertrophy and prostatitis with two prior TURP procedures. This patient also elected to undergo HBO. No patient developed a urethral stricture or any grade 4 or 5 GU toxicity. Logistic regression also identified no patient characteristics associated with an increased risk of late grade 3 GU toxicity.

**Table 5:** **Patients with late grade 3 hematuria**.

Patient	Age	Prior transurethral resection of the prostate	Antiplatelets	Time to bleeding (months)	Areas of RT changes on cystoscopy	Fulguration
1^a^	65	No	Aspirin (81 mg)	18, 36	Left lateral wall, trigone, and posterior bladder neck	Yes
2	68	No	N/A	18	Base of bladder	Yes
3	73	No	Aspirin (unknown dose)	24	All areas except dome	Yes
4	79	No	N/A	30	Bladder neck and posterior wall	Yes

*No patient was on anticoagulation therapy*.

*^a^Patient elected to have hyperbaric oxygen therapy*.

## Discussion

This study aimed to assess the safety of performing IMRT with SBRT boost for unfavorable clinically localized prostate cancer. SBRT boost was chosen for this study due to the potential radiobiologic benefits of hypofractionation (29) as well as the ease of the treatment modality, especially for the elderly prostate cancer patient population.

Intensity-modulated radiation therapy plus SBRT boost was generally well tolerated with minimal acute toxicity (Tables 2 and 3). Cumulative late ≥grade 2 and ≥grade 3 GU toxicities were observed in 40 and 6% of patients, respectively. It should be noted that the seemingly high rate of grade 2 GU toxicity was due to use of alpha-antagonists or corticosteroids for transient irritative or obstructive symptoms. In fact, the prevalence of any grade 2 GU toxicity was 10–16% after the 3-month time period (Table 3). It is encouraging that only 4% of patients developed hematuria requiring fulguration, and only 2% developed retention requiring a TURP. In comparison, published brachytherapy boost studies have reported late GU toxicity rates of 8–31% (≥grade 2) and 3–18% (≥grade 3) (Table 6) (14–16, 30). Importantly, the ASCENDE-RT trial reported a cumulative late ≥grade 3 GU toxicity rate of 18% at 6 years for patients undergoing a LDR boost, most commonly due to urethral strictures, urinary retention, or incontinence (30). Our 6% cumulative rate of clinically significant late GU toxicity is lower than that reported in this trial, though longer follow-up will be necessary to confirm our results. Other institutions using the SBRT boost technique have reported a 0–2.3% rate of late ≥grade 3 GU toxicities, which are comparable to our results reported here (20–23).

**Table 6:** **Summary of late ≥grade 2 or 3 toxicities reported for various techniques, including IMRT + SBRT boost**.

Author	Institution/trial	Technique	Dose (Gy)	Median follow-up (years)	Pts	Gr 2 genitourinary (GU) (%)	Gr 3 GU (%)	Gr 2 gastrointestinal (GI) (%)	Gr 3 GI (%)
Zelefsky et al. (6)	MSKCC	IMRT/IGRT	86.4	2.8	186	10.4	–	1.0	–
		IMRT/no IGRT			190	20	–	1.6	–

Michalski et al. (31)	Radiation Therapy Oncology Group (RTOG) 0126	3D-CRT	79.2	4.6	491	13.4	2.5	22	5.1
		IMRT		3.5	257	7.8	1.9	15.1	2.6

Mariados et al. (32)	PIVOT	IMRT/No spacer	79.2	1.25	73	4.2	–	1.4	–
		IMRT/spacer			149	6.8		0	

King et al. (33)	UCLA/Stanford	SBRT (5 fxns)	36.25	2.7	67	8.8	3.5	2	0

Chen et al. (27)	Georgetown	SBRT (5 fxns)	35–36.25	2.3	100	31	1	1	0

Khor et al. (13)	Melbourne, Australia	HDR boost (3 fxns) + EBRT	19.5 + 46	5	344	16.8^a^	11.8^a^	–	–

Hoskin et al. (15)	UK	HDR boost (2 fxns) + EBRT (13 fxns)	17 + 35.75	7.1	110	31^b^	–	7^b^	–

Hsu et al. (16)	RTOG 0321	HDR boost (2 fxns) + EBRT	19 + 45	2.5	112	7.1	2.7	2.7	0.9

Rodda et al. (30)	ASCENDE-RT	LDR boost + EBRT	115	6.5	198	–	18	–	9
		EBRT	78		200		8		4

Katz and Kang (21)	Winthrop	SBRT boost (3 fxns) + 3D-CRT	(19 to 21) + 45	5	45	4.6	2.3	13.3	–

Lin et al. (20)	Taiwan	SBRT boost (3 fxns) + VMAT	21 + 45	3.5	41	3–11^c^	0	0	0

Anwar et al. (23)	UCSF	SBRT boost (2 fxns) + SIB	(9.5 to 10.5) + 45	3.6	48	27	2	0	0

Paydar et al. (24)	Georgetown	SBRT boost + IMRT	19.5 + (45 to 50.4)	4.2	108	40	6	12	1

*IMRT, intensity-modulated radiation therapy; IGRT, image-guided radiation therapy; 3D-CRT, 3D-conformal radiation therapy; SBRT, stereotactic body radiation therapy; HDR, high dose rate; LDR, low dose rate; EBRT, external beam radiation therapy; VMAT, volumetric arc therapy; SIB, simultaneous integrated boost*.

*^a^Urethral stricture rates*.

*^b^Severe toxicity per the Dische scale*.

*^c^0–11% toxicity rates in late follow-up period with cumulative rates not reported*.

Our study reported overall modest rates of GI toxicity, with a 12% cumulative incidence of late ≥grade 2 GI toxicity, 7% late ≥grade 2 rectal bleeding, and 1% late grade 3 bleeding. In comparison, we have previously reported a 1.5% rate of late ≥grade 2 rectal bleeding with SBRT alone (34). Other studies have reported a somewhat lower 1–3% rate of late ≥grade 2 rectal bleeding with brachytherapy boost (16, 30). Moreover, 16 (15%) of our patients were noted to have telangiectasias, 3 of which were multiple confluent telangiectasias (VRS grade 3). No VRS grade 3 telangiectasias were previously noted with SBRT alone (34).

Despite overall higher rates of GI and GU toxicity compared to SBRT alone, a sample DVH of the combined SBRT and IMRT plans for one patient (Figure 1) demonstrates that the bladder volume receiving 55 and 70 Gy and the rectal volumes receiving 50 and 70 Gy in 2 Gy equivalents are well below the Radiation Therapy Oncology Group recommendations (35). This suggests that the wide IMRT margins and the resulting near-maximal dose at the bladder neck and anterior rectal wall likely contribute to bleeding events, and future dosimetric studies will need to define the appropriate dose-constraints for patients treated with this approach.

Previously, we reported that IMRT with SBRT boost resulted in minimal impact on long-term bowel QOL (19), a finding which is seemingly discordant with late toxicity results shown here. A similar phenomenon has been seen following IMRT monotherapy (31, 36). Several explanations are possible. For example, the most common toxicities—rectal bleeding, hematuria, and urinary obstruction—were transient and resolved by the following time point (37). Also, bleeding likely renders a less bothersome impact on QOL than frequency and urgency (38). Lastly, effects on QOL are reported as temporal changes in mean scores derived from the expanded prostate cancer index composite-26 questionnaire while the CTCAE toxicity scoring system focuses on individual uncommon events (39).

Analysis of such uncommon events is still necessary, since telangiectasias or ulcers may be a precursor lesion for a fistula. While a small percentage of telangiectasias progress to fistulas and often do so after multiple invasive procedures (40, 41), such a late toxicity can nonetheless be devastating for a patient. Thus, it is encouraging that only three of our patients required two APC procedures, with no reportable fistulas to date. Though fistulas most often develop within the first 3 years (40), longer follow-up is still necessary to detect a potential late occurrence.

What is the minimum follow-up time to assess late toxicity after prostate SBRT such that clinically meaningful events are captured fully without undue delay in reporting these important outcomes? For the majority of patients, rectal bleeding occurred at the 1- to 1.5-year time point and resolved after one to two APC procedures, suggesting that a 3-year median follow-up likely captures most rectal bleeding events. However, hematuria occurred starting at 18 months post-treatment and continued to present as late as 36 months, emphasizing the necessity of long-term follow-up for such patients. While no fistulas or strictures of the urethra have been observed, longer follow-up may be necessary to reveal such toxicity as well.

Our encouraging rates of GI and GU toxicity are consistent with results from other institutions using this modality (20–23), though minor differences in technique do exist. For instance, the three largest published series included pelvic lymph nodes in the external beam radiotherapy portion (20–23) and utilized a 3D-conformal (21, 22), volumetric arc therapy (20), or simultaneous integrated boost (23) technique for dose delivery. It should be emphasized that no overall survival benefit has been demonstrated thus far for the treatment of pelvic nodes in high-risk disease (42, 43), and inclusion of pelvic nodes remains a matter of controversy. Furthermore, different dose-fractionation schemas as well as PTV margins were used for the SBRT portion in each study. For instance, Anwar et al. utilized PTV margins of 0 mm posteriorly and 2 mm elsewhere (23). Such smaller margins may have contributed to the lower rates of overall toxicity, with only one late grade 3 GU and no grade 3 GI toxicities reported.

These low toxicity rates question the use of larger PTV margins or IMRT at all. Even with dose-escalated external beam radiotherapy for clinically localized prostate cancer, most failures occur locally within the prostate or adjacent seminal vesicles (44). However, surgical pathology studies report a median ECE of 0.5–2.4 mm and a 4–5 mm margin necessary to cover ≥90% of the ECE (45–48). Our treatment planning studies have furthermore demonstrated potential under-dosing of the posterior prostate without adequate PTV margin (49). Thus, the appropriate planning technique remains an ongoing debate, and long-term outcomes will identify the optimal combinatorial approach as well as target volume.

One approach to maintaining adequate posterior margins while reducing the risk of rectal bleeding is to place a tissue equivalent spacer in the perirectal space prior to treatment. This approach can increase percent target coverage of the PTV while simultaneously reducing the rectal volume receiving near-maximal dose (50). In fact, a recent prospective trial demonstrated a statistically significant reduction in late rectal toxicity from 7 to 2% with the use of rectal spacers in the setting of IMRT (32). An improvement in long-term bowel and 6-month urinary QOL decline was also shown with the use of spacers (32). At our institution, we have recently initiated rectal spacer use for high-risk patients receiving IMRT with SBRT boost for a potential reduction in late rectal bleeding. If rectal spacers effectively prevent rectal bleeding in all patients, 14 patients would require spacer placement to prevent one cauterization event for bleeding, assuming a 7% bleeding rate. While the impact on QOL would undoubtedly be positive, the cost-effectiveness of this therapy remains a matter of debate (51).

## Conclusion

Fiducial-guided IMRT with SBRT boost is a promising new treatment option for men with unfavorable prostate cancer. Early results suggest encouraging biochemical response, minimal impact on long-term QOL, and low toxicity. These data provide a basis for the design of a phase III clinical trial.

## Author Contributions

IP is the lead author, who participated in data collection, data analysis, manuscript drafting, table/figure creation, and manuscript revision. AP participated in data collection, manuscript drafting, and table/figure creation. RC and JK participated in data collection. TY aided in clinical data collection. EB is a dosimetrist who developed the majority of patients’ IMRT treatment plans and contributed to the dosimetric data analysis and interpretation. SL is the dosimetrist who developed the majority of patients’ SBRT treatment plans and contributed to the dosimetric data analysis and interpretation. AS and KH aided in patient enrollment and collection of clinical data. SS is a senior author who collected the dosimetric data, participated in its analysis, and helped draft the manuscript. AD, JL, and TK are senior authors who aided in drafting the manuscript. SC was the principal investigator who initially developed the concept of the study and the design, aided in data collection, and drafted and revised the manuscript. All the authors read and approved the final manuscript.

## Conflict of Interest Statement

SC serves as a clinical consultant to Accuray Inc. The Department of Radiation Medicine at Georgetown University Hospital receives a grant from Accuray to support a research coordinator. The other authors declare that they have no competing interests.

## Abbreviations

3D-CRT, 3D-conformal radiation therapy; ADT, androgen deprivation therapy; APC, argon plasma coagulation; CCI, Charlson comorbidity index; CTC, common terminology criteria; CTCAEv.4, common terminology criteria for adverse events version 4.0; CTV, clinical target volume; DVH, dose–volume histogram; ECE, extracapsular extension; EBRT, external beam radiation therapy; EPIC-26, expanded prostate cancer index composite-26; GI, gastrointestinal; GU, genitourinary; HBO, hyperbaric oxygen; HDR, high dose rate; IGRT, image-guided radiation therapy; IMRT, intensity-modulated radiation therapy; LDR, low dose rate; MRI, magnetic resonance imaging; NCI, National Cancer Institute; NPO, Non-Per *Os*; OAR, organs at risk; PSA, prostate-specific antigen; PTV, planning target volume; QOL, quality of life; RTOG, Radiation Therapy Oncology Group; SBRT, stereotactic body radiation therapy; SIB, simultaneous integrated boost; TURP, transurethral resection of the prostate; VMAT, volumetric arc therapy; VRS, Vienna Rectoscopy Score.
